# Forecasting Obesity and Type 2 Diabetes Incidence and Burden: The ViLA-Obesity Simulation Model

**DOI:** 10.3389/fpubh.2022.818816

**Published:** 2022-04-05

**Authors:** Roch A. Nianogo, Onyebuchi A. Arah

**Affiliations:** ^1^Department of Epidemiology, Fielding School of Public Health, University of California, Los Angeles (UCLA), Los Angeles, CA, United States; ^2^California Center for Population Research (CCPR), Los Angeles, CA, United States; ^3^Department of Statistics, Division of Physical Sciences, UCLA College, Los Angeles, CA, United States; ^4^Research Unit for Epidemiology, Department of Public Health, Aarhus University, Aarhus, Denmark

**Keywords:** agent-based model, obesity, type 2 diabetes, simulation, prediction

## Abstract

**Background:**

Obesity is a major public health problem affecting millions of Americans and is considered one of the most potent risk factors for type 2 diabetes. Assessing future disease burden is important for informing policy-decision making for population health and healthcare.

**Objective:**

The aim of this study was to develop a computer model of a cohort of children born in Los Angeles County to study the life course incidence and trends of obesity and its effect on type 2 diabetes mellitus.

**Methods:**

We built the Virtual Los Angeles cohort—ViLA, an agent-based model calibrated to the population of Los Angeles County. In particular, we developed the ViLA-Obesity model, a simulation suite within our ViLA platform that integrated trends in the causes and consequences of obesity, focusing on diabetes as a key obesity consequence during the life course. Each agent within the model exhibited obesity- and diabetes-related healthy and unhealthy behaviors such as sugar-sweetened beverage consumption, physical activity, fast-food consumption, fresh fruits, and vegetable consumption. In addition, agents could gain or lose weight and develop type 2 diabetes mellitus with a certain probability dependent on the agent's socio-demographics, past behaviors and past weight or type 2 diabetes status. We simulated 98,230 inhabitants from birth to age 65 years, living in 235 neighborhoods.

**Results:**

The age-specific incidence of obesity generally increased from 10 to 30% across the life span with two notable peaks at age 6–12 and 30–39 years, while that of type 2 diabetes mellitus generally increased from <2% at age 18–24 to reach a peak of 25% at age 40–49. The 16-year risks of obesity were 32.1% (95% CI: 31.8%, 32.4%) for children aged 2–17 and 81% (95% CI: 80.8%, 81.3%) for adults aged 18–65. The 48-year risk of type 2 diabetes mellitus was 53.4% (95% CI: 53.1%, 53.7%) for adults aged 18–65.

**Conclusion:**

This ViLA-Obesity model provides an insight into the future burden of obesity and type 2 diabetes mellitus in Los Angeles County, one of the most diverse places in the United States. It serves as a platform for conducting experiments for informing evidence-based policy-making.

## Introduction

Obesity is a major public health problem affecting millions of Americans with two in three adults and one in three children considered overweight or obese (1). This condition disproportionately affects lower-income minority and disadvantaged groups (1) giving rise to health disparities. Obesity has been on the rise for the past few decades (1, 2) despite ongoing prevention efforts warranting its description as a pervasive and complex phenomenon (3, 4). As a result, the obesity epidemic has been suggested to result from the complex interplay between individual and environmental factors and behaviors (3, 4). This complexity is clearly seen when considering the socio-ecological framework (5) and exemplified by the fact that our individual behaviors can be influenced by our past behaviors (6), the neighborhood we live in (7) and the people around us (8).

Obesity (and overweight) is considered one of the most potent risk factors for type 2 diabetes (9). Almost 80–90% of type 2 diabetes patients are overweight or obese. This is alarming as type 2 diabetes is a disabling disease that imposes considerable burden on individuals, families, communities and the health system. The total direct medical and indirect expenditures attributable to diabetes in the U.S. amounted to ~$245 billion in 2012 (10).

To model obesity and forecast its future, researchers have suggested using complex methods (3, 4). One such method is an agent-based model—a computer representation of the real world (11, 12) where researchers and policymakers can run experiments *in silico* to evaluate the impact of potential interventions by simulating counterfactual scenarios (13). An example of such a virtual world is represented by the Coronary Heart Disease Policy Model developed to forecast and address coronary heart disease incidence, mortality and cost (14). Another prominent model is the Archimedes diabetes model (15), which was built to address clinical problems and questions around diabetes and modeled after several randomized controlled trials. In the present study, we chose to model our virtual world after that of Los Angeles County, California, for its high population density, its ethnic diversity (16), its rising rates of obesity and its marked racial/ethnic disparities in obesity (17).

In addition, modeling approaches that provide different and complementary insights on how changes in individual and environmental risk factors could affect disease rates in the future in a recent birth cohort are needed. Therefore, we set up a discrete-time modeling approach that will incorporate trends in individual and environmental risk factors in the hopes of evaluating their joint effects, at critical life stages, on future obesity or diabetes status in a recent birth cohort (13).

The overarching goal of this study was to develop an agent-based simulation model of a cohort of children born in Los Angeles County and followed into adulthood to study the life-course development of obesity and of its effects on diabetes mellitus. Specifically, we aimed to forecast and study the life course incidence and trends of obesity and its effect on type 2 diabetes mellitus risk. This synthetic cohort could serve as a platform for conducting *in silico* experiments and testing hypothetical public health interventions to inform evidence-based clinical and population-health decision- and policy-making (13, 18).

## Methods

We developed the ViLA–Obesity model, a stochastic, dynamic, discrete-time, agent-based model informed by various data sources and calibrated to the population of Los Angeles County in California to explore the incidence and trends in obesity and type 2 diabetes.

### Description of the ViLA Simulated Population and Overview of the ViLA-Obesity Simulation Model

According to the 2010 US Census, Los Angeles County was inhabited by 9,818,605 individuals who lived in 2,346 census tracts (19). In this model, as it is the case in some other studies (20), we considered a census tract to represent a neighborhood. We simulated 235 neighborhoods with 418 inhabitants per neighborhood for a total simulated population of 98,230, which represented a 100th of the Los Angeles County (LAC) total population (Supplementary Table 1). Simulated individuals in the model are referred to as agents. In this closed cohort, each agent was born in a specific neighborhood and was simulated from birth (aged 0–1 year, i.e., *time* = *0*) to middle adulthood (aged 60–65 years, i.e., *time* = *9*) in 10 discrete time steps representing critical life stages (Supplementary Table 2). At each time step the agent's age is simulated using a uniform distribution bounded within the specific critical life stages (Supplementary Table 2).

**Figure 1:**
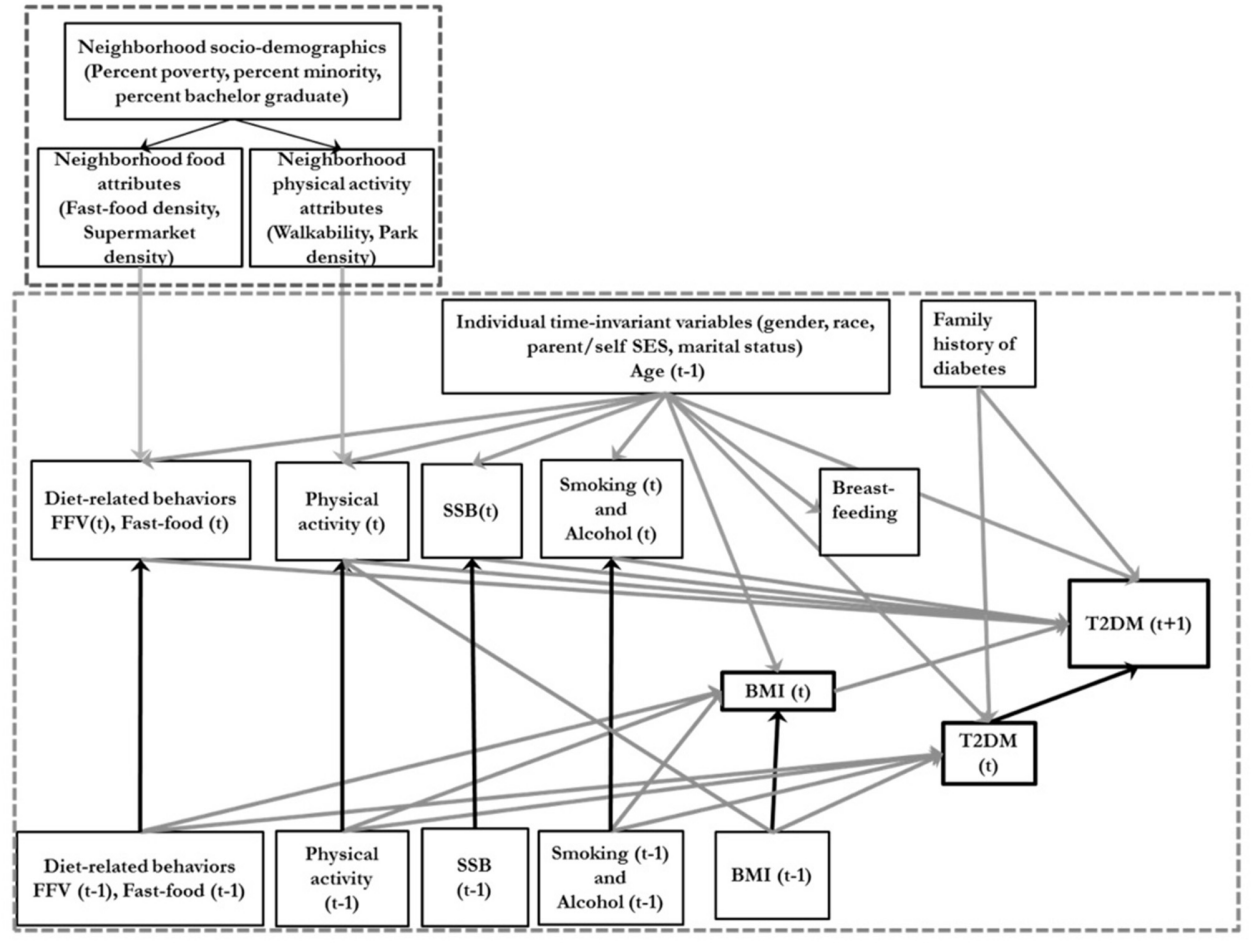
Conceptual directed acyclic diagram underlying the data-generating process. SSB, sugar-sweetened beverage consumption; BMI, body mass index; FFV, Fresh fruit and vegetable consumption; T2DM, type 2 diabetes; Ado, Adolescence. T is an index of time. The smaller dotted square represents the neighborhood variables and the larger dotted square represents the individual level variables.

**Figure 2:**
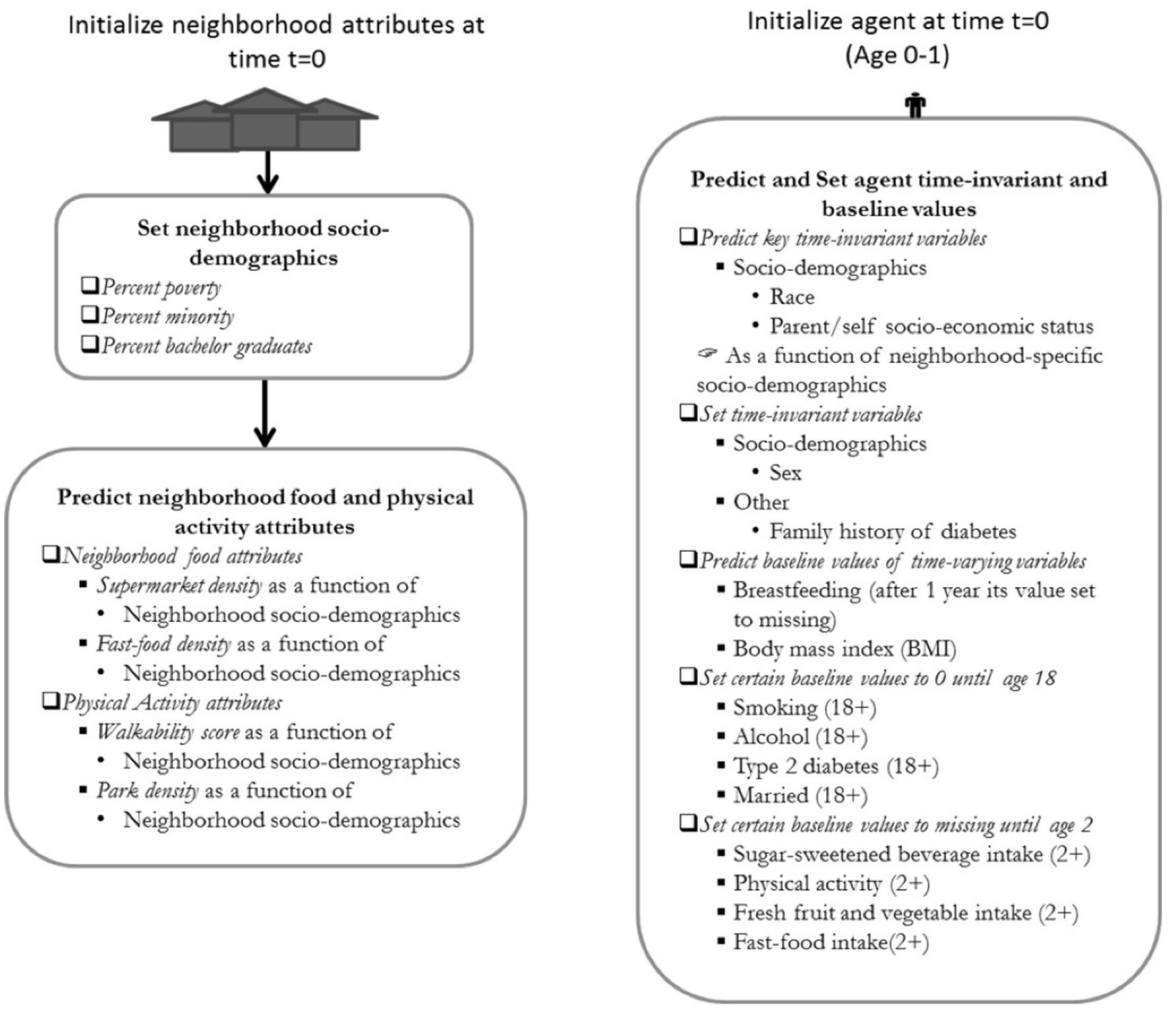
Model initialization diagram of the ViLA-Obesity model. The neighborhood attributes are first initialized at time t=0 and subsequently followed by the initialization of the agent. The neighborhood food and physical activity attributes are predicted as a function of neighborhood socio-demographics. The individual time-invariant variables are set to their baseline values or predicted from socio-demographic variables.

**Figure 3:**
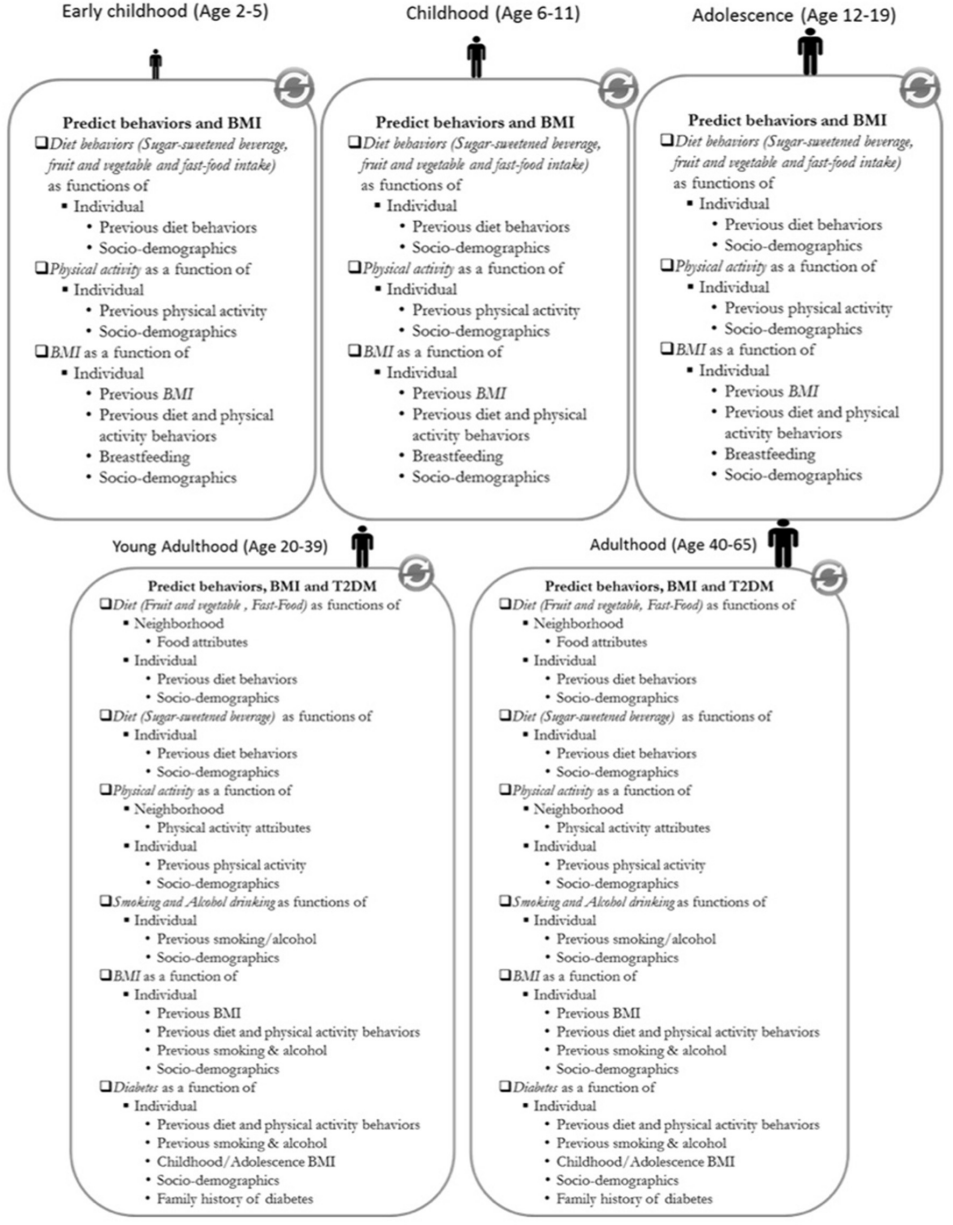
Model execution diagram of the ViLA-Obesity model. BMI, body mass index; T2DM, type 2 diabetes; SES, Socio-economic status. Individual behaviors are predicted based on the agent's socio-demographics, previous behavior and depending on the behavior neighborhood characteristics. The body mass index and type 2 diabetes are predicted based on the agent's socio-demographics, previous behaviors, and previous body mass index in the case of body mass index.

ViLA-Obesity represents a simulation model or suite within our ViLA platform. It integrates trends in the causes and consequences of obesity, focusing on diabetes as a key obesity consequence during the life course. During the simulation, each agent exhibited obesity- and diabetes-related healthy and unhealthy behaviors [e.g., sugar-sweetened beverage consumption (SSB), physical activity, smoking], gained/lost weight and developed type 2 diabetes with a certain probability dependent on the agent's current state (Figures 1–3). We calculated and reported age-specific incidence, cumulative incidence, prevalence and average incidence rate of obesity and diabetes. To calculate the incidence measures, we considered the first-time diagnosis of obesity or type 2 diabetes among at-risk individuals. All data preparation and analysis and Monte Carlo simulation were also done in SAS 9.4 software (Cary, NC).

### Data Sources and Parameters

- Proportions, means and standard deviations:

The parameters for the individual-level socio-demographics and those of the neighborhood-level socio-demographics were obtained from the American Community Survey (ACS) (Supplementary Table 4). The individual-level race and income group were derived respectively from the neighborhood-specific race percentage and percent below federal poverty level (FPL). The proportions, means and standard deviations of the individual-level exposures and outcomes [breastfeeding, SSB, physical activity, fast-food consumption and fruit and vegetable consumption, smoking, alcohol consumption, body mass index (BMI), type 2 diabetes] were obtained from the California Health Interview Survey (CHIS) (21), the Centers for Disease Control and Prevention (CDC) (22), the World Health Organization (WHO) (Supplementary Table 5).

- Parameters for effect and association measures:

These regression coefficients were taken from various sources detailed in the Supplementary Table 3. For clarity, we defined three levels of evidence. “Evidence level 1” parameters are directly taken, in this order of preference, from published systematic reviews and meta-analyses, randomized control trial studies or cohort studies. “Evidence level 2” parameters are directly taken from cross-sectional studies from the peer-reviewed literature. “Evidence level 3” parameters are computed (indirectly obtained) by our research team using merged publicly and privately available data [e.g., American Community Survey, National Establishment Time-Series (NETS), Walkscore.com, WHO, National Health and Nutrition Examination Survey (NHANES) and the Los Angeles County Health and Nutrition Examination Survey (LAHANES) (21–25). Ideally, all parameters would be coming from “evidence level 1” but because most studies do not report on the relationships between covariates such as age, sex, race, socio-economic status [SES], and the outcome and between the covariates and the exposures, we identified other sources of evidence (Supplementary Tables 6–10).

### Model Specification

#### Agent

Each simulated agent had three domains of attributes. The first domain was the agent's socio-demographics [i.e., age, sex, socio-economic status (SES), race/ethnicity and marital status] representing the individual's inherent susceptibility which was not allowed to change (i.e., time-invariant variables) with the exception of age. We assumed that individuals born in a certain SES group will remain in that group until the end of the simulation (i.e., inherit their parents' SES) and that agents could only get married after their 18th birthday (Supplementary Table 4). The second domain was the agent's behaviors and was divided into: (i) dietary behaviors (breastfeeding, fast-food consumption, SSB, fresh fruit, and vegetable consumption); (ii) physical activity behaviors (moderate-to-vigorous physical activity) and (iii) other behaviors (smoking, alcohol consumption) (Supplementary Table 5). The last domain was the agent's outcomes (BMI, and type 2 diabetes status).

Agents were only allowed to engage in smoking, alcohol consumption and develop type 2 diabetes after their 18th birthday. Both behavior and outcome domains were considered time-varying variables. For children aged 0–19, we defined overweight and obesity using the WHO BMI Z-score international child cutoffs (26). We calculated BMI Z-scores using CDC's SAS codes (27). Based on the WHO growth charts, a child with a BMI Z-score (BMIz) < -2 was classified as underweight; a BMIz ≥ −2 but <1 was classified as normal-weight; a BMIz ≥ 1 but < + 2 was classified as overweight and a BMIz ≥ 2 was classified as obese (28).

Similarly, an adult with a BMI <18.5 was classified as underweight; a BMI ≥ 18.5 but <25 was classified as normal-weight; a BMI ≥ 25 but <30 was classified as overweight and a BMI ≥ 30 was classified as obese (29).

#### Neighborhood Environment

The neighborhood where the agents dwelled had three domains. The first domain was the neighborhood socio-demographics encompassing the proportion of individuals who self-identified as non-White, the proportion of individuals living below the federal poverty level (FPL) and the proportion of individuals who had a bachelor's degree or higher. The data for this domain were obtained from the American Community Survey [ACS] (19). The second domain was the neighborhood physical activity opportunities that comprise the neighborhood walkability and access to parks. The data for the second domain were obtained from Walkscore.com (30), the National Establishment Time-Series (NETS) (31) and Wolch et al. (30). The third domain was the neighborhood food environment comprising the supermarket and the fast-food density. The data for the third domain were obtained from NETS (31) (see Supplementary Table 4 for more details).

#### Conceptual Model, Equations, and Decision Rules

The decision rules underlying this model were mainly based on mathematical equations. Completely exogenous variables in this model were few and limited to individual- and neighborhood-level socio-demographics. Except at birth (time *t* = 0), all behavior equations (e.g., SSB, physical activity) had a common form whereby the dependent variable would be a function of the following: intercept, lagged version of the dependent variables and socio-demographics. Likewise, the outcome equations (e.g., BMI, type 2 diabetes) had in addition to the previous ones listed all age-specific behaviors (e.g., SSB, physical activity, and smoking). Linear and logistic regressions were used for modeling continuous and binary dependent variables, respectively. Accordingly, the inverse of the link functions used in the regression modeling were used for simulation (i.e., identity and expit functions respectively). The neighborhood environment and its attributes are first simulated, then agents with their attributes by time period are simulated within neighborhoods. These will engender a change in BMI and will subsequently affect diabetes risk. Most endogenous variables allow for time-dependency (i.e., previous behavior affecting future behavior). Features of feedback were also allowed. For instance, when BMI changed, it affected subsequent ability to exercise which subsequently affected future BMI and so on (32). A detailed description of the equation structure are presented in the Supplementary Table 11.

### Model Calibration, Verification, and Validation

We undertook several iterative steps to build the ViLA-Obesity model. These included calibration, validation, and verification. Of note, *calibration* is the process through which we assign input parameters within the model and ensure that the predicted model output is close to that of the observed data (ideally using training data if available or the entire data if not). Evaluating whether calibration worked within one's own data could also be seen as an internal validation procedure. *Validation (*or sometimes external validation), on the other hand, strives to ensure that the predicted model output (ideally using a training data if available or using the entire data if not) is close that of the observed data (ideally using a test data or using observed data from a different period if not). *Verification* is a process that involves different techniques such as structured code walk-throughs to check for model consistency and errors and makes sure that the model does what it is intended to do (33, 34).

#### Model Verification

In the model verification step, we used structured code walk-through to check for model consistency and errors throughout the modeling process in an iterative fashion.

#### Model Calibration and Internal Validation

We first obtained parameters (i.e., proportions, means, standard deviations of each variable, and the regression coefficients relating any two variables) from multiple studies and datasets. Many commonly used external validation techniques (35) could not be used here because we did not have a base cohort in Los Angeles that followed individuals from birth to adulthood and which studied our exposures and outcomes of interests. In other words, we could not externally validate our model. Nevertheless, we used a “calibration-in-the-large” technique to calibrate and internally validate our model (35). In brief, the “calibration-in-the-large” is a calibration whereby one ensures that the mean predicted outcome equals the mean observed outcome [i.e., mean(*Y*_*predicted*_) = mean(*Y*_*observed*_)] through the fine tuning of the intercept (35), or other coefficient. The finding of the equality mean(*Y*_*predicted*_) = mean(*Y*_*observed*_) ensured the internal validity of the model testifying that there was agreement between the observed data and our model predictions (i.e., internal validation). From a practical standpoint, after we have assigned the parameters in our equation models (see Supplementary Table 11, e.g., relative risks obtained from the three levels of evidence, etc.), we sought to find and finetune the remaining parameters, that is, those that could not be otherwise obtained directly from the literature. There were two such parameters: intercepts and feedback parameters (i.e., coefficients reflecting the relationship between current behavior or outcome to previous behavior and outcome, all else equal). As such, we defined a calibration objective function as the Mean Absolute Error (MAE) between the predicted and observed variable mean or prevalence. Once the objective function has been defined, we used a grid search strategy to find the appropriate parameters of interest. Parameter values that minimized the objective function were selected to parametrize the model. This was done sequentially starting from birth (aged 0–1 year, i.e., *time* = *0*) to middle adulthood (aged 60–65 years, i.e., *time* = *9*) in 10 discrete time steps. Furthermore, after the whole model parametrization, we evaluated whether our calibration (internal validation) was successful by (1) plotting our simulated and observed outcome means and proportions over time for each behavior [e.g., sugar-sweetened beverage (SSB)] and outcome (e.g., body mass index) and (2) computing the variance explained, *R*^2^, between the simulated and observed data for each behavior and outcome over time. As such, we internally validated our model on the basis of its ability to the predict observed outcomes. To extend the model to other populations, we could adjust our intercepts to match the site-specific observed prevalence (35).

## Results

### Calibration and Internal Validation

**Figure 4:**
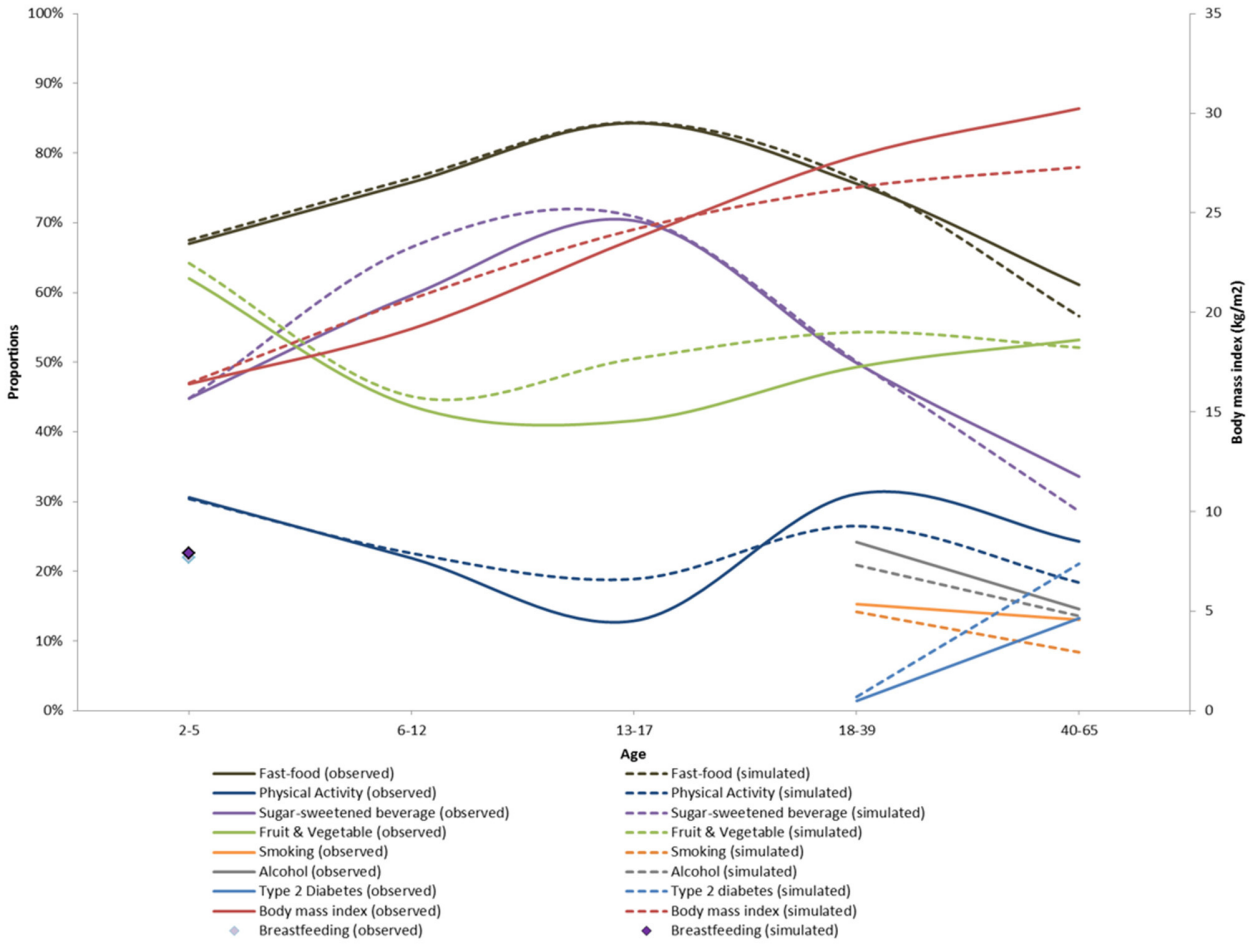
Calibration of the ViLA-Obesity model. This figure depicts the results of the model calibration. It compares the observed (plain lines) to the simulated data (dotted lines).

Figure 4 shows the simulated and observed means and proportions by age groups. Our simulation results broadly matched the age-specific means and proportions from CHIS 2009. However, there were some small but notable departures from the observed data for physical activity, fresh fruit and vegetable consumption, smoking and diabetes prevalence. This can also be seen with the computed *R*^2^ which was high (>0.9) for body mass index, sugar sweetened beverage, fresh fruits and fast-food consumption and moderate (>0.6) for fresh fruits and vegetables and physical activity. The *R*^2^ for exclusive breastfeeding, smoking, alcohol and type 2 diabetes could not be computed because of the low number of data points available (see Supplementary Table 12 for details).

### Trends in Obesity and Type 2 Diabetes

**Figure 5:**
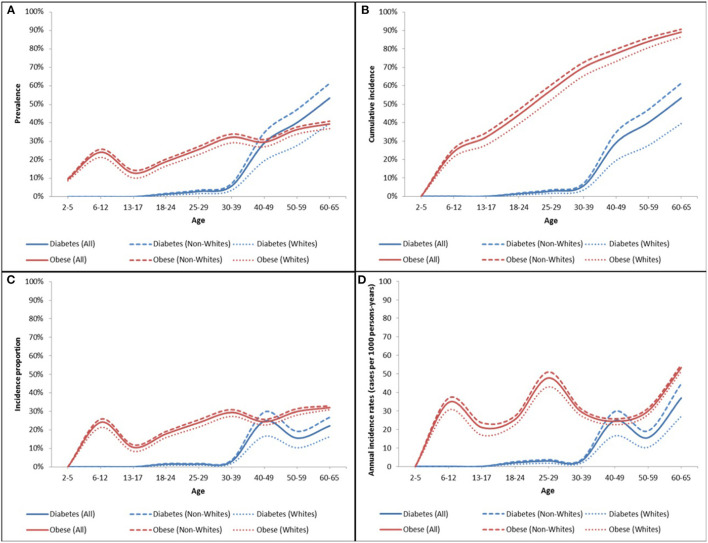
Obesity and type 2 diabetes prevalence **(A)**, cumulative incidence **(B)**, age-specific incidence proportion **(C)**, and annual incidence rates **(D)** in the ViLA-Obesity model. The incidence measures were calculated for first-time diagnosis of obesity or type 2 diabetes among at-risk individuals (i.e., without the diagnosis).

Figure 5 depicts the overall and racial subgroup trends (incidence and prevalence) in obesity and type 2 diabetes over time in the ViLA-Obesity model.

We found that the obesity age-specific incidence proportion was generally increasing from about 10% to about 30% across the individual life span with two notable peaks at age 6–12 and 30–39. Likewise, the age-specific incidence proportion of type 2 diabetes increases from <2% at age 18–24 to reach a peak of about 25% at age 40–49.

The prevalence of obesity was highest in childhood with about 25% of children considered obese between the age of 6 and 12 years. During adulthood, the prevalence of obesity rose to reach a maximum of 40% at the end of follow-up at age 60–65 years.

Compared to Whites, the incidence and prevalence of obesity and type 2 diabetes were generally higher among the non-White subpopulation. There were marked disparities in the prevalence of type 2 diabetes compared to that of obesity. The racial disparity gap in the prevalence of type 2 diabetes was greatest during middle adulthood but that in the prevalence of obesity was small but more uniform across ages.

### Trends in Drivers of Health Behaviors

**Figure 6:**
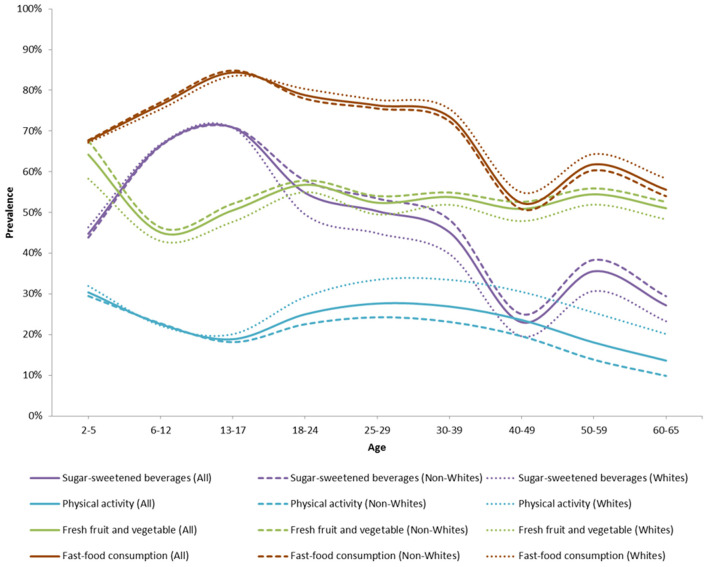
Proportion of obesity- and type 2 diabetes-related health behaviors over time in the ViLA-Obesity model. This figure highlights the health behaviors prevalence across the population.

Figure 6 shows the overall and racial subgroup trends in key health behaviors. The consumption of fast-food was generally high and decreasing with age. It was highest during childhood and adolescence with ~75–85% of children and adolescents consuming fast-foods more than one time per week. The consumption of sugar-sweetened beverage was also generally high and decreasing with age. It was highest during childhood and adolescence with ~60–70% of children and adolescents consuming more than one 12-oz drink of SSB per day. Engaging in moderate-to-vigorous physical activity was generally low and decreasing with age. It was lowest during adolescence with only about 20% of adolescents engaging in moderate-to-vigorous physical activity. The consumption of fresh fruits and vegetables was fairly constant over time. It was lowest during childhood with only about 40–50% of children aged 6–12 consuming more than five servings of fruit and vegetables per day. About one out of five individuals were breastfed for 6 months or longer during their 1st year of life.

### Cumulative Incidence and Average Incidence Rate of Obesity and Type 2 Diabetes in the ViLA-Obesity Model

Table 1 presents the cumulative incidence and average incidence rates of obesity and type 2 diabetes in the ViLA-Obesity model.

**Table 1:** Incidence rates and cumulative incidence of obesity and type 2 diabetes in the ViLA-Obesity model (*n* = 98,230).

**ALL**				
	**Obesity (2–65)**	**Obesity childhood (2–17)**	**Obesity adulthood (18–65)**	**Type 2 diabetes adulthood (18–65)**
**Total number**	98,230	98,230	98,230	98,230
**Events**	87,625	31,544	79,606	52,426
**Person-years (py)**	3,183,963	1,415,891	2,847,196	4,099,783
**Incidence rate (per 1,000 py)**	27.5 (27.3, 27.7)	22.3 (22.0, 22.5)	28.0 (27.8, 28.2)	12.8 (12.7, 12.9)
**Cumulative incidence**	89.2% (89.0%, 89.4%)	32.1% (31.8%, 32.4%)	81.0% (80.8%, 81.3%)	53.4% (53.1%, 53.7%)
**Whites**				
**Total number**	35,862	35,862	35,862	35,862
**Events**	31,072	10,023	28,067	14,162
**Person-years (py)**	1,245,482	523,022	1,090,448	1,571,629
**Incidence rate (per 1,000 py)**	25.0 (24.7, 25.2)	19.2 (18.8, 19.5)	25.7 (25.4, 26.0)	9.0 (8.9, 9.2)
**Cumulative incidence**	86.6% (86.3%, 87.0%)	28.0% (27.5%, 28.4%)	78.3% (77.8%, 78.7%)	39.5% (38.9%, 40.0%)
**Non-whites**				
**Total number**	62,368	62,368	62,368	62,368
**Events**	56,553	21,521	51,539	38,264
**Person-years (py)**	1,938,481	892,869	1,756,748	2,528,154
**Incidence rate (per 1,000 py)**	29.2 (28.9, 29.4)	24.1 (23.8, 24.4)	29.3 (29.1, 29.6)	15.1 (15.0, 15.3)
**Cumulative incidence**	90.7% (90.4%, 90.9%)	34.5% (34.1%, 34.9%)	82.6% (82.3%, 89.3%)	61.4% (61.0%, 61.7%)

*The incidence measures were calculated for first-time diagnosis of obesity or type 2 diabetes among at-risk individuals*.

#### Type 2 Diabetes

The 48-year risk or cumulative incidence of type 2 diabetes in the ViLA-Obesity model was 53.4% (95% CI: 0.53.1%, 0.53.7%) and the average incidence rate of type 2 diabetes was about 13 cases per 1,000 person-years (95% CI: 12.7, 12.9) for adults aged 18–65 years.

#### Obesity

The 16-year risk or cumulative incidence of obesity was 32.1% (95% CI: 31.8%, 32.4%) and the average incidence rate of obesity was about 22 cases per 1,000 person-years (95% CI: 22.0, 22.5) for children aged 2–17 years. The 48-year risk or cumulative incidence of obesity was 81% (95% CI: 80.8%, 81.3%) and the average incidence rate of obesity was about 28 cases per 1,000 person-years (95% CI: 27.8, 28.2) for adults aged 18–65 years.

## Discussion

The purpose of this study was to build an agent-based model of a cohort of children born in Los Angeles County and followed from birth into adulthood in order to study the life course development of obesity and of its effects on diabetes mellitus. This virtual cohort would then serve as a platform for conducting *in silico* experiments and testing hypothetical public health interventions to inform evidence-based clinical decision- and policy-making (13, 18).

Our findings suggest that the incidence and prevalence of obesity and type 2 diabetes within the ViLA-Obesity model were generally high and increasing during the life span. The prevalence of obesity was highest during childhood and among individuals in their 30's while the prevalence of type 2 diabetes started rising among individuals in their 40's. In addition, one in three children and adolescents and four in five adults will become obese before age 65 and one in two adults will develop type 2 diabetes before age 65 in the simulated cohort. There were some racial differences in the prevalence and incidence of obesity and type 2 diabetes. The non-White subpopulation experienced higher proportions of individuals who became obese or developed type 2 diabetes at any point in time throughout the 64-year follow-up compared their White counterparts. The presence of such racial disparities in obesity and type 2 diabetes has been well-documented in Los Angeles (17, 36).

Furthermore, our results also suggested that the proportion of individuals engaging in moderate-to-vigorous physical activity and consuming at least five servings of fresh fruit and vegetables was generally low while the proportion of individuals consuming fast-food and drinking sugar-sweetened beverages was generally high within the simulated cohort. There were also some racial differences among these obesity-related health behaviors. Among the non-White subpopulation, there was a lower proportion of individuals who engaged in moderate-to-physical activity, and a higher proportion of individuals who drank more than one sugar-sweetened beverage a day compared to their White counterparts. In contrast, among the White subpopulation, there was a lower proportion of individuals who ate fresh fruit and vegetables and a higher proportion of individuals who ate fast-food more than once per week compared to their non-White counterparts.

This study provided a unique perspective of the development of obesity and type 2 diabetes among individuals who would have been followed from birth into adulthood in Los Angeles. This approach allowed us to simultaneously appreciate the aging effect on and forecast the future burden of obesity and type 2 diabetes within a birth cohort between 2009 and 2074 (i.e., 2009+65), something that has seldom been done in the literature. In addition, our modeling approach provides different and complementary insights on how disease rates will change in the future in a recent birth cohort. Specifically, our discrete-time modeling approach will allow researchers to see how current or future obesity or diabetes burden could reflect the joint and cumulative effects of prior and current environmental and individual exposures at critical life stages. In other words, as individual and environmental risk factors change over time, so will the trends in obesity and diabetes be expected to change.

Importantly, unless done for calibration purposes, one should be cautious when comparing our estimates to past and projected prevalence and incidence of obesity and diabetes. In fact, many trend estimates are based on cross-sectional data which typically reflect a given period effect and averaged across several age-groups and birth cohorts (37, 38). Nevertheless, these past and projected trends remain important for gauging the current and potential future state of obesity and diabetes in Los Angeles and the US. For instance, in 2011, the prevalence of obesity was 22.4% among children and 23.6% among adults (17) and the prevalence of diabetes was 9.9% (36) among adults in Los Angeles County. In the absence of projection studies in Los Angeles County, one can look to regional and national projection data to better appreciate the burden of disease attributable to obesity and type 2 diabetes. In fact, the UCLA Health forecasting tool, a simulation model that simulated individual life course among California's adult population, predicted that the obesity and type 2 diabetes prevalence will reach 30.8 and 9.93% respectively by 2020 in their baseline scenario (39). In addition, other projection studies based on nationally representative data found that the prevalence of impaired glucose tolerance could reach 15% by 2048 (40) and that the prevalence of obesity could reach 51.1% by the year 2030 (41). The latter study also predicted that 80, 90, and 100% of Americans will become obese by the year 2072, 2087, and 2102, respectively and that the non-White subpopulation may reach those levels sooner compared to Whites (41). Interestingly, when using the linear annual rate of increase reported in that study and the prevalence of obesity among adults in Los Angeles in 2011, we estimated that the projected prevalence of obesity in 2074 would be ~67%. A study of the growth trajectory, which used a simulation model, also found that about 57.3% could become obese by the age of 35 (42). Lastly, the predicted life-time risk of diagnosed diabetes from age 20 was estimated to be about 40% for men and women in a nationally representative sample (43). All of these projections reflect similar alarming trends as suggested by our model and their insights warrant immediate action to reverse or slow the epidemic in the US and in Los Angeles County in particular.

This study has several limitations. First, the calibration and validation of the ViLA-Obesity model were suboptimal in the absence of a base cohort in Los Angeles that followed individuals from birth to adulthood and studied our exposures and outcomes of interests. Nevertheless, we used age-group-specific means and proportions from publicly available data (i.e., CHIS) representing whenever available the population of Los Angeles County in 2009. This has some limitations since it does not allow one to disentangle the cohort/secular trend effects from the age effects. As such, we have assumed that the cohort/secular trend effect would be smaller relative to that of the age effect since we are simulating each individual as they age over time within the simulated cohort. Our results may reflect at the very least the age effect but could also reflect age and cohort effects. In addition, as cross-sectional data typically include people who are more likely to have chronic conditions such as diabetes, the use of such data for our calibration could result in the overestimation of the measures of occurrence within our simulation. Nevertheless, in the absence of longitudinal data, using age-group specific data in a specific year appears to be a better alternative than using repeated cross-sectional data to calibrate our model since the latter would not allow one to disentangle age and period effects. Second, while we have incorporated relevant obesity-related environmental exposures, we did not account for the possibility of residual social network effect in this iteration of the model. While there have been some suggestions that obesity can spread through social networks (i.e., induction or person-to-person spread) (8), other authors have demonstrated that such effects may be the result of confounding by contextual exposures (e.g., food environment, built-environment) (44). These authors concluded that after properly accounting for environmental exposures, the social network effects in obesity almost vanished (44). This finding, however, did not mean that peer support could not enhance the effectiveness of certain prevention efforts (45). We hope to explore the added insights gained from incorporating social network effects in the next iteration of the model. Third, the ViLA-Obesity model represented a simplified version of the Los Angeles County population in that the simulated cohort was closed (that is agents could not drop out, die, experience a competing risk, beget children, move in and out of the cohort). This will likely result in an overestimation of the incidence and prevalence measures. Future iteration of the model will incorporate competing risk in the data generating process. Fourth, using larger age categorization for calibration could result in suboptimal model calibration. We chose this approach since the regression parameters obtained from internal data analysis and to some extent from the literature was generally obtained for similar larger age categorization (most likely because of sample size consideration). Fifth, it is possible that the inclusion of large number of parameters and predictors in the model could add some additional uncertainty in the estimates produced by the model. We have included information on both individual factors as well as environmental factors because we intended to evaluate the impact of several interventions including single and combined interventions at the individual level and at environmental level at different critical life stages. Nevertheless, although the model has recently been used to evaluate impacts of obesity related-interventions (13), we believe such models should continue to undergo refinement through continuous validation and calibration as data and methods improve and new applications are found. In addition, the model was built to represent a 100th of the actual population of Los Angeles and agents were only allowed to engage in certain behaviors (e.g., smoking, alcohol consumption, and develop type 2 diabetes) after their 18th birthday.

### Uses of the ViLA Modeling Suite

The current model will be kept up to date to reflect current trends and changes in trends in individual and environmental factors over time. In addition, we hope to incorporate additional outcomes including but not limited to cardiovascular diseases and cancer. The ViLA-Obesity suite has been used to evaluate single and combined (i.e., joint and cumulative) impact several known and hypothetical interventions that target individual and or environmental factors (13). For instance, the Los Angeles County Department of Public Health (LAC/DPH) in collaboration with the Center for Disease Control and Prevention (CDC) implemented from 2010 to 2012 several interventions to curb the obesity epidemic such as the “Community Putting Prevention to Work (CPPW)” with the RENEW project (Renew Environments for Nutrition, Exercise, and Wellness). The project “sought to implement policy, systems, and environmental changes to improve nutrition, increase physical activity, and reduce obesity, especially in disadvantaged communities” (46). As an initial modeling endeavor, we proposed to evaluate the long-term effects of individual-level dietary interventions (e.g., breastfeeding promotion, and reduction of sugar-sweetened beverages) and environmental physical activity-related interventions (e.g., increasing access to parks and recreations and designing pedestrian-friendly communities) on obesity and diabetes incidence in the ViLA cohort (13). Generally, to evaluate the effectiveness of an intervention, we would contrast the projected incidence and prevalence under say a hypothetical scenario where we would “alter” the exposure status to the desired level (intervention course) to the projected incidence and prevalence under the natural course (no interventions) (13).

## Conclusion

We developed and validated a virtual cohort representing Los Angeles County wherein we explored the development of obesity and diabetes from birth to adulthood. Our findings suggest that the incidence and prevalence of obesity and type 2 diabetes within the ViLA-Obesity model were generally high and increasing with age during the individual life span. In this virtual Los Angeles, one in three children and adolescents and four in five adults will become obese before age 17 and age 65 respectively and one in two adults will develop type 2 diabetes before age 65. We also noted the presence of racial disparities in obesity, type 2 diabetes, and obesity-related behaviors. This virtual cohort serves as a platform for conducting *in silico* experiments and testing hypothetical public health interventions to inform evidence-based clinical decision and policymaking. This study illustrates the usefulness of simulations like agent-based models in forecasting the burden of disease within a population over time to support the need for effective interventions.

## Data Availability Statement

The relevant data used in this study are included in the article/Supplementary Material, further inquiries about the full list of data citations can be directed to the corresponding author/s.

## Author Contributions

RN participated in the study conception, design, analysis, and wrote the first draft of the article. OA supervised and participated in the study conception, design, analysis, reviewed, and revised the manuscript. All authors provided critical input and insights into the development, writing of the article, and approved the final manuscript as submitted.

## Funding

RN was supported by a Burroughs Wellcome Fellowship and the Dissertation Year Fellowship from UCLA. OA was partly supported by grant R01-HD072296-01A1 from the Eunice Kennedy Shriver National Institute of Child Health and Human Development. In addition, RN benefited from facilities and resources provided by the California Center for Population Research at UCLA (CCPR), which receives core support (R24-HD041022) from the Eunice Kennedy Shriver National Institute of Child Health and Human Development (NICHD).

## Conflict of Interest

The authors declare that the research was conducted in the absence of any commercial or financial relationships that could be construed as a potential conflict of interest.

## Publisher's Note

All claims expressed in this article are solely those of the authors and do not necessarily represent those of their affiliated organizations, or those of the publisher, the editors and the reviewers. Any product that may be evaluated in this article, or claim that may be made by its manufacturer, is not guaranteed or endorsed by the publisher.
