# Supplementary material for: Forecasting Obesity and Type 2 Diabetes Incidence and Burden: The ViLA-Obesity Simulation Model
Source: Front Public Health. 2022 Apr 5;10:818816. doi: 10.3389/fpubh.2022.818816 (PMC9016163; doi:10.3389/fpubh.2022.818816)
Supplement: Supplementary file 1 [file Data_Sheet_1.pdf]

Forecasting Obesity and Type 2 Diabetes Incidence and Burden: The ViLA-Obesity Simulation Model

Roch A. Nianogo & Onyebuchi A. Arah

Supplemental materials

Contents

Table 1 Los Angeles County, California actual and simulated population sizes 3
Table 2 Life period, time-points and age-group 3
Table 3 General outline for the data sources of parameters for effect and association measures .. 4
Table 4 Input parameters for the distribution of individual and neighborhood time-invariant variables 5
Table 5 Input parameters for the distribution of individual time-varying variables 6
Table 6 Input parameters for the effect/association between individual-level exposures and individual-level outcomes ('Evidence level 1' parameters) 8
Table 7 Input parameters for the effect/association between neighborhood-level exposures and individual-level exposures ('Evidence level 2' parameters) 10
Table 8 Input parameters for the effect/association between neighborhood-level demographics and neighborhood-level exposures ('Evidence level 3' parameters) 11
Table 9 Input parameters for the effect/association between individual-level covariates and individual-level exposures, NHANES 1999-2014 ('Evidence level 3' parameters) 12
Table 10 Input parameters for the effect/association between individual-level covariates and individual-level outcome, NHANES 1999-2014, ('Evidence level' 3 parameters) 14
Table 11 Simplified equation structure underlying the model 15
Table 12 Evaluation of the model calibration using the estimated R^2 16

Input parameter estimation and assignment

We have mathematically converted some estimates obtained from the literature to fit the model needs using simulations where necessary. These instances include but are not limited to: converting a mean difference into a risk ratio or an odds ratio; converting a rescaled effect into an appropriate scaled effect; converting an estimate obtained using a continuous predictor into an estimate that would be obtained using a categorical version of the predictor; converting proportions obtained from a contingency table into an association measure; converting a weight difference into a BMI difference by dividing it by a common age-group specific height.

To obtain regression coefficients between the individual-level exposures and the individual-level outcomes (BMI, type 2 diabetes), we used parameters from ‘evidence level 1’ parameters (**Supplemental Table 6**). To obtain regression coefficients between the neighborhood-level variables (walkability, park access, supermarket density, fast-food density) and the individual-level variables (physical activity, fast-food consumption, fruit and vegetable consumption), we used parameters from our defined ‘evidence-level 2’ parameters (**Supplemental Table 7**). To obtain regression coefficients between the neighborhood-level socio-demographics (percent non-White, percent below FPL, percent bachelor graduates) and the neighborhood-level exposures (walkability, park access, supermarket density, fast-food density), we used parameters from our defined ‘evidence-level 3’ parameters (**Supplemental Table 8**). Lastly, to obtain the regression coefficients between the individual-level covariates (e.g. sex, race, marital status, SES) and the individual-level outcomes (BMI, type 2 diabetes) and between the individual-level covariates and the individual-level exposures (breastfeeding, SSB, physical activity, fast-food consumption and fruit and vegetable consumption), we used parameters from our defined ‘evidence-level 3’ parameters (**Supplemental Table 9 & Supplemental Table 10**).

Table 1 Los Angeles County, California: actual and simulated population sizes

	Actual population[2]	***Simulated population***
Number of census tracts	2,346	235
Population Density	4,185.25	418
Number of census tracts × population density	9,818,605	98,230

Note: The simulated population represents a 100th of the LAC total population rounded to the nearest ones. Population density was calculated by dividing the total population size by the number of census tracts.

Table 2 Life period, time-points, and age-group

**Life period**	**Time**	**Age group**
Birth	0	0-1
Early Childhood	1	2-5
Middle Childhood	2	6-12
Adolescence	3	13-17
Young Adulthood	4	18-24
Young Adulthood	5	25-29
Young Adulthood	6	30-39
Middle Adulthood	7	40-49
Middle Adulthood	8	50-59
Middle Adulthood	9	60-65

Table 3 General outline for the data sources of parameters for effect and association measures

Relations	Variables	Evidence type
**Individual-level exposures to individual-level outcomes**	Individual-level exposures<br>• Breastfeeding<br>• Sugar-sweetened beverage<br>• Physical activity<br>• Fast-food<br>• Fruit and vegetable<br>Individual-level outcomes<br>• BMI<br>• Type 2 diabetes mellitus	Evidence-level 1<br>RCTs, Systematic Reviews, Meta-analyses, cohort studies<br>➔ From the literature
**Neighborhood-level exposures to individual-level exposures**	Neighborhood-level exposures<br>• Walkability<br>• Park access<br>• Supermarket density<br>• Fast-food density<br>Individual-level exposures<br>• Physical activity<br>• Fast-food consumption<br>• Fruit and vegetable consumption	Evidence-level 2<br>Cross-sectional studies<br>➔ From the literature
**Neighborhood-level socio-demographics to neighborhood-level exposure relations**	Neighborhood-level socio-demographics<br>• Percent non-White<br>• Percent below federal poverty level<br>• Percent bachelor graduates<br>Neighborhood-level exposures<br>• Walkability<br>• Park access<br>• Supermarket density<br>• Fast-Food density	Evidence-level 3<br>Merged publicly available survey data (ACS, NETS, WalkScore data, NHANES)<br>➔ From our analysis
**Covariates–exposures Covariates–outcomes relations**	Individual-level covariates<br>• Sex<br>• Race<br>• Marital status<br>• Low-income status<br>• Family history diabetes<br>Individual-level exposures<br>Individual-level outcomes	

Table 4 Input parameters for the distributions of individual and neighborhood time-invariant variables

Variables	Values: Mean (SE) or %	Sources	Years	Distribution	Bound
**Neighborhood-level parameters**					
Percent non-White	0.72 (0.26)	ACS, 2014 [2]	0-65	Normal	[0,1]
Percent below federal poverty level	0.19 (0.13)	ACS, 2014 [2]	0-65	Normal	[0,1]
Percent bachelor graduates graduate or above	0.28 (0.21)	ACS, 2014 [2]	0-65	Normal	[0,1]
**Individual-level parameters**					
Sex (Male vs Female)	Male: 49%	ACS, 2014 [2]	0-65	Bernoulli	[0,1]
Race (White vs Non-White)	Derived from neighborhood specific percent non-White	ACS, 2014 [2]	0-65	Bernoulli	[0,1]
Income group (Below or at FPL vs. Above FPL)	Derived from neighborhood specific percent below the federal poverty level	ACS,2014 [2]	0-65	Bernoulli	[0,1]
Marital Status (Married vs Not Married)	Married: 44%	ACS, 2014 [2]	18-65	Bernoulli	[0,1]

SE: Standard error; FPL: Federal Poverty Level; ACS: American community survey

Table 5 Input parameters for the distribution of individual time-varying variables

Variables	Values (by age group)	Sources	Years	Distribu-tion	Bound
Breastfeeding<br>Breastfed exclusively for six months or more	0-1 year: 22%	CDC[3]	0-1	Bernoulli	[0,1]
Fast-food consumption<br>Ate fast-food more than one times (1 to 4) in past week	2-5 years: 67%<br>6-12 years:76%<br>13-17 years: 84%<br>18-39 years: 76%<br>40-65 years: 61%	CHIS, 2009 [4]	2-65	Bernoulli	[0,1]
Moderate-to-vigorous physical activity<br>Physically active at least one hour per day for 7 days [age 2-17 years]<br>Moderate physical activity >=30 min/day for 5 days (including walking): [age 18-65 years]	2-5 years: 31%<br>6-12 years:22%<br>13-17 years: 13%<br>18-39 years: 31%<br>40-65 years: 24%	CHIS, 2009 [4]	2-65	Bernoulli	[0,1]
Sugar-sweetened beverage consumption<br>Drank one or more glasses of soda or other sugary drinks yesterday	2-5 years: 67%<br>6-12 years: 76%<br>13-17 years: 84%<br>18-39 years: 76%<br>40-65 years: 61%	CHIS, 2009 [4]	2-65	Bernoulli	[0,1]
Fresh fruit and vegetable consumption<br>Ate five or more servings of fruits and vegetables	2-5 years: 62%<br>6-12 years: 44%<br>13-17 years: 42%<br>18-39 years: 49%<br>40-65 years: 53%	CHIS, 2009 [4]	2-65	Bernoulli	[0,1]
Smoking<br>Current smoker	18-39 years: 24%<br>40-65 years: 15%	CHIS, 2009 [4]	18-65	Bernoulli	[0,1]
Alcohol consumption<br>Binge drinking	18-39 years: 76%<br>40-65 years: 61%	CHIS, 2009 [4]	18-65	Bernoulli	[0,1]
Type 2 diabetes<br>Yes	18-39 years: 1.4%<br>40-65 years: 13.3%	CHIS, 2009 [4]	18-65	Bernoulli	[0,1]
Body mass index (kg/m2)	0-1: 16.33 (1.49)<br>2-5 years: 16.41 (1.99)<br>6-12 years: 19.18 (4.66)<br>13-17 years: 23.69 (5.73)	WHO[5] LAHANES, 2011[6]	0-65	Normal	

18-39 years:	27.85 (6.90)
40-65 years:	30.23 (6.90)

Table 6 Input parameters for the effect/association between individual-level exposures and individual-level outcomes ('Evidence level 1' parameters)

Exposure variable	Dependent variable	Point Estimate & 95%CI	Model covariates	Study	Notes
Exclusive breast-feeding	Body mass index	MD=-0.14 (-0.26, -0.02)	Age, gender, birth weight, BMI of the mother and educational level of the mother	(7)	
Moderate-to-vigorous physical activity	Body mass index	MD=-0.43 (-0.63, -0.23)	Age, sex	(8)	
	Type 2 diabetes	RR= 0.65 (0.59, 0.71)	N/A	(9)	
Sugar-sweetened beverage consumption	Body mass index	MD=0.08 (0.03, 0.13)	N/A	(10)	
	Type 2 diabetes	RR= 1.28 (1.12; 1.46)	Adiposity, within person variation, sociodemographic variables, clinical factors (family history of diabetes or prevalent diseases), and lifestyle factors, including diet	(11)	
Fresh fruit and vegetable consumption	Body mass index	MD=-0.13	Baseline age, BMI and change in the following lifestyle variables: smoking status, physical activity, hours of sitting or watching TV, hours of sleep, fried potatoes, juice, whole grains, refined grains, fried foods, nuts, whole-fat dairy, low-fat dairy, sugar-sweetened beverages, sweets, processed meats, non-processed meats, trans fat, alcohol, and seafood	(12)	Outcome was weight in kg but was converted to BMI by dividing weight in kg by a common US adult height (1.645 meter). Exposures were fruits and vegetables separately but was combined to obtain one exposure (fruit and vegetable consumption/ day)
	Type 2 diabetes	RR=0.96 (0.91, 1.01)	smoking, alcohol, total energy intake, BMI, physical activity, FHDM, education and other dietary factors	(13)	

Table 6 Input parameters for the effect/association between individual-level exposures and individual-level outcomes ('Evidence level 1' parameters) (continued)

Exposure variable	Dependent variable	Point Estimate & 95%CI	Model covariates	Study	Notes
Fast-food consumption	Body mass index	MD=0.66	age, sex, education, site, baseline weight height, alcohol, TV, physical activity	(14)	Outcome was weight in kg in Blacks and Whites separately but was converted to a common BMI by dividing weight in kg by a common US adult height (1.645 meter)
	Type 2 diabetes	HR/RR=1.51 (1.25, 1.83)		(15)	Exposure was consumption of processed red meat
Body mass index in childhood	Type 2 diabetes	OR=1.24		(16)	The effect was expressed in in terms of odds ratio per standard deviation BMI but authors stated that the reported "[odds ratio] was approximately equivalent to a 24% increase in odds of diabetes per kg/m2 in BMI"
Body mass index	Moderate-to-vigorous physical activity	OR=0.96 (0.94, 0.98)	Smoking habits, sex, sedentary lifestyle at age 41, and changes in BMI from ages 41 to 44 and 44 to 46	(1)	Outcome was sedentary lifestyle so we took the inverse to express the effect of BMI on physical activity<br>The OR presented is an annualized OR

SE: Standard error; MD: Mean difference; OR: Odds ratio; HR: Hazard ratio; RR: risk ratio; BMI: body mass index

Table 7 Input parameters for the effects/associations between neighborhood-level exposures and individual-level exposures ('Evidence level 2' parameters)

**Predictors**	**Dependents**	**Point estimates**	**Model covariates**	**Study**	**Notes**
Neighborhood supermarket (per square mile)	Fruits and vegetables consumption	RR=1.33 (1.05, 1.69)	Age, race, sex, per capita annual income	(17)	Actual outcome: Alternative Healthy Eating Index
Neighborhood Fast-food density (#outlets/mile)	Fast-food consumption	OR=1.11 (0.98, 1.26)	Age, education, per capita HH income, race, sex, site	(18)	Outcome is fast-food ≥ 1 times/week within 1 mile vs. never
Neighborhood walkability	Physical activity	OR=1.74 (1.51, 2.01)	Age, gender, education, BMI, days in the U.S., and habitual physical activity level in Cuba	(19)	Outcome is whether engaged in purposive walking last week Original walk score exposure has been dichotomized (i.e. walk score >=70) and odds ratio for engaging in purposeful walking re-adjusted
Access to Parks	Physical activity	OR=1.50 (1.06, 2.13)	Age, gender, education, children <18 in home, SES	(20)	Outcome: ≥ 6 walking sessions/week totaling >180 minutes. Exposure: Very good access to public open spaces (i.e. = top quartile of access) vs. very poor access to public open spaces; Access to public open spaces is defined on the basis of distance, attractiveness and size

CI: Confidence interval; SE: Standard error; MD: Mean difference; OR: Odds ratio; HR: Hazard ratio; RR: risk ratio

Table 8 Input parameters for the effect/association between neighborhood-level demographics and neighborhood-level exposures ('Evidence level 3' parameters)

Dependents	Model predictors and standard errors	Source	Notes
High neighborhood walkability	Intercept: log-odds(0.0171*)<br>Percent Non-White: OR=20<br>Percent below FPL: OR=6.70<br>Percent bachelor graduates: OR=41.21	ACS, 2014[2]<br>Walkscore.com	High neighborhood walkability was defined as having a Walk score ≥ 70 (Very walkable to walker's paradise) vs. poor walkability (i.e., walk score < 70, Car-dependent to somewhat walkable)
Park Access	Intercept: log-odds (0.5055*)<br>Predominantly non-white: OR=1.85<br>Predominantly below FPL: OR=1.32	Wolch et al. [21]	We used the contingency tables in the article to construct estimate<br>Access to parks was defined as the percent of population living within a quarter-mile buffer
Fast-food density	Intercept: 0<br>Percent non-white: MD=0.99<br>Percent below FPL: MD=5.86<br>Percent bachelor graduates: MD:1.40<br>Standard error: 3.49	ACS, 2014 [2]<br>NETS[22]	
Supermarket density	Intercept: -0.40<br>Percent non-white: MD=0.51<br>Percent below FPL: MD=3.74<br>Percent bachelor graduates: MD=1.12<br>Standard error: MD=2.38	ACS, 2014[2]<br>NETS [22]	

** = calibrated intercept; FPL: Federal poverty level; OR: Odds ratio; MD: Mean difference; Predominantly White was defined as having percent non-White >=75%; predominantly poor was defined as having a percent below federal poverty level>=40% as done in* Wolch et al. [21]

Table 9 Input parameters for the effect/association between individual-level covariates and individual-level exposures, NHANES 1999-2014 ('Evidence level 3' parameters)

		**Predictors**												
**Outcomes**		Inter-cept*	Lagged* (OR)	Age (OR)	Male (OR)	Non-White (OR)	Low-Income (OR)	Low-Income (OR)	Married (OR)	BMI (OR)	EnvPRK (OR)	EnvWLK (OR)	EnvSMD (OR)	EnvFFD (OR)
Birth	EBF	0.231	·	·	0.98	1.00	0.9	0.9	·	·	·	·	·	·
Early child-hood	MVPA	0.260	·	1.05	1.49	0.88	1.11	1.11	·	·	1.00	1.00	·	·
	FFD	0.646	·	1.05	1.08	1.00	1.10	1.10	·	·	·	·	·	1.00
	FFV	0.586	·	0.98	0.93	1.50	1.24	1.24	·	·	·	·	1.00	·
	SSB	0.296	·	1.34	1.21	0.90	2.26	2.26	·	·	·	·	·	·
Middle childhood	MVPA	0.254	0.869	0.97	1.25	1.01	1.09	1.09	·	0.96[&]	1.00	1.00	·	·
	FFD	0.639	2.203	1.02	0.98	1.10	0.99	0.99	·	·	·	·	·	1.00
	FFV	0.600	0.198	1.02	1.18	1.39	1.37	1.37	·	·	·	·	1.00	·
	SSB	0.318	9.679	1.15	1.46	1.02	1.40	1.40	·	·	·	·	·	·
Adolescence	MVPA	0.221	0.069	0.98	1.33	0.93	0.90	0.90	·	0.96[&]	1.5	1.74	·	·
	FFD	0.637	4.759	1.02	0.95	1.10	1.07	1.07	·	·	·	·	·	1.00
	FFV	0.600	0.198	1.02	1.18	1.29	1.34	1.34	·	·	·	·	1.00	·
	SSB	0.358	8.004	1.15	1.52	1.02	0.37	0.37	·	·	·	·	·	·

** The intercept and lagged variable regression coefficients have been obtained from our calibration algorithm to match the observed means and prevalence.*
NHANES: National health and nutrition examination survey 1999-2014; OR: Odds ratio; EBF: Exclusive breastfeeding (i.e., exclusively breastfed ≥ 6months) ; FFD: Fast-food consumption (i.e., ate fast-food ≥ 1 time in past week) ; MVPA: Moderate-to-vigorous physical activity (i.e., engage in moderate-to-vigorous physical activity); SSB: Sugar-sweetened beverage consumption (i.e., drank ≥ 1 glasses of soda or sugary drinks); FFV: Fresh fruit and vegetable consumption; SMK: Smoking (i.e., current smoking); ALC: Alcohol consumption (i.e., binge drank alcohol the past month); EnvWLK: Environment or neighborhood walkability; EnvPRK: Environment or neighborhood park access; EnvSMD: Environment or neighborhood supermarket density; EnvFFD: Environment or neighborhood fast-food density;
[&]These odds ratios were taken from the literature ('evidence level 1') whereas the others are computed from NHANES 1999-2014[(23)].

Table 9 Input parameters for the effect/association between individual-level covariates and individual-level exposures, NHANES 1999-2014 ('Evidence level 3' parameters) (continued)

		Predictors												
		Intercept*	Lagged* (OR)	Age (OR)	Male (OR)	Non-White (OR)	Low-Income (OR)	Low-Income (OR)	Married (OR)	BMI (OR)	EnvPRK (OR)	EnvWLK (OR)	EnvSMD (OR)	EnvFFD (OR)
Young adult-hood	MVPA	0.174	19.688	0.98	1.17	0.69	0.57	0.57	1.06	0.96[&]	1.50	1.74	·	·
	FFD	0.659	1.448	0.98	1.32	0.89	1.15	1.15	0.87	·	·	·	·	1.11
	FFV	0.604	0.079	0.99	1.73	1.38	1.39	1.39	1.21	·	·	·	1.33	·
	SSB	0.395	1.020	0.97	2.42	1.44	1.68	1.68	0.86	·	·	·	·	·
	ALC	0.220	0.80	0.97	1.94	0.87	1.56	1.56	0.74	·	·	·	·	·
	SMK	0.220	1.04	0.97	1.60	0.47	1.94	1.94	0.54	·	·	·	·	·
Adult-hood	MVPA	0.130	19.298	0.98	1.17	0.57	0.58	0.58	1.21	0.96[&]	1.50	1.74	·	·
	FFD	0.651	0.869	0.97	1.25	0.87	1.14	1.14	0.83	·	·	·	·	1.11
	FFV	0.570	0.098	0.99	1.72	1.41	1.36	1.36	1.18	·	·	·	1.33	·
	SSB	0.371	0.427	0.96	2.38	1.48	1.62	1.62	0.80	·	·	·	·	·
	ALC	0.070	21	0.96	2.33	21	2.10	2.10	0.62	·	·	·	·	·
	SMK	0.060	21	0.98	1.67	21	2.10	2.10	0.44	·	·	·	·	·

** The intercept and lagged variable regression coefficients have been obtained from our calibration algorithm to match the observed means and prevalence. NHANES: National health and nutrition examination survey 1999-2014; OR: Odds ratio; EBF: Exclusive breastfeeding (i.e. exclusively breastfed ≥ 6months) ; FFD: Fast-food consumption (i.e., ate fast-food ≥ 1 time in past week); MVPA: Moderate-to-vigorous physical activity (i.e., engage in moderate-to-vigorous physical activity); SSB: Sugar-sweetened beverage consumption (i.e., drank ≥ 1 glasses of soda or sugary drinks); FFV: Fresh fruit and vegetable consumption; SMK: Smoking (i.e., current smoking); ALC: Alcohol consumption (i.e. Binge drank alcohol the past month); EnvWLK: Environment or neighborhood walkability; EnvPRK: Environment or neighborhood park access; EnvSMD: Environment or neighborhood supermarket density; EnvFFD: Environment or neighborhood fast-food density;*

[&]These odds ratios were taken from the literature ('evidence level 1') whereas the others are computed from NHANES 1999-2014[(23)].

Table 10 Input parameters for the effects/associations between individual-level covariates and individual-level outcome, NHANES 1999-2014, (‘Evidence level’ 3 parameters)

		Birth	Early Childhood	Middle Childhood	Adolescence	Young Adulthood		Adulthood	
		**Outcomes**							
		BMI (MD)	BMI (MD)	BMI (MD)	BMI (MD)	BMI (MD)	T2DM (OR)	BMI (MD)	T2DM (OR)
**Predictors**	Intercept	15.74	16.24	17.067	18.89	20.559	0.00002*	22.68	0.00032*
	Lagged	·	0.006	-0.070	0.35	0.19	·	0.28	·
	BMI_Ado	·	·	·	·	·	1.24[&]	·	1.24[&]
	Age	0.4947	-0.10	0.86	0.56	0.18	1.12	0.02	1.07
	Male	0.4389	0.20	-0.19	-0.60	-0.68	0.99	-1.05	1.45
	Non-White	0.15	0.15	0.72	0.90	0.88	1.74	0.36	2.14
	Low-income	0.11	0.11	0.32	0.37	0.63	1.55	0.13	1.59
	Married	·	·	·	·	-0.06	1.21	-0.61	1.14
	BMI	·	·	·	·	·	1.11	·	1.11
	Exclusively breastfed ≥ 6months	·	-0.14[&]	·	·	·	·	·	·
	Engage in moderate-to-vigorous physical activity	·	-0.43[&]	-0.43[&]	-0.43[&]	-0.43[&]	0.65[&]	-0.43[&]	0.65[&]
	Ate fast-food ≥ 1 times in past week	·	0.66[&]	0.66[&]	0.66[&]	0.66[&]	1.51[&]	0.66[&]	1.51[&]
	Eat ≥ 5 fresh fruits and vegetables/day	·	-0.13[&]	-0.13[&]	-0.13[&]	-0.13[&]	0.96[&]	-0.13[&]	0.96[&]
	Drank ≥ 1 glasses of soda or sugary drinks	·	0.08[&]	0.08[&]	0.08[&]	0.08[&]	1.28[&]	0.08[&]	1.28[&]
	Current smoker	·	·	·	·		1.25	-2.15	1.13
	Binge drank alcohol the past month	·	·	·	·		1.50	0.62	1.26
	Has family history of type 2 diabetes	·	·	·	·	·	4.07	·	3.57
	Standard deviation	1.49	1.994	4.657	5.733	6.9	·	6.9	·
	Minimum	10.76	12.58	12.40	13.30	15.5	·	8.9	·
	Maximum	23.56	33.20	46.100	50.70	62.9	·	72.9	·

**Calibrated intercept; OR: Odds ratio; MD: Mean difference*

[&]*These parameters were taken from the literature (‘evidence level 1’) whereas the others were computed from NHANES 1999-2014.*

Table 11 Simplified equation structure underlying the model

$SSB_t = B(1,\ expit\ (Int_{SSB} + \beta_{SSBt-1} * ssb_{t-1} + \beta_{AGE} * age_t + \beta_{MALE} * male + \beta_{NONWHITE} * nonWhite + \beta_{LOWINC} * lowinc + \beta_{MARRIED} * married\ ))$
$FFD_t = B(1,\ expit\ (Int_{FFD} + \beta_{FFDt-1} * ffd_{t-1} + \beta_{AGE} * age_t + \beta_{MALE} * male + \beta_{NONWHITE} * nonWhite + \beta_{LOWINC} * lowinc + \beta_{MARRIED} * married + \beta_{EnvFFD} * EnvFFD\ ))$
$MVPA_t = B(1,\ expit\ (Int_{MVPA} + \beta_{MVPAt-1} * mvpa_{t-1} + \beta_{AGE} * age_t + \beta_{MALE} * male + \beta_{NONWHITE} * nonWhite + \beta_{LOWINC} * lowinc + \beta_{MARRIED} * married + \beta_{EnvPRK} * EnvPRK + \beta_{EnvWLK} * EnvWLK))$
$FFV_t = B(1,\ expit\ (Int_{FFV} + \beta_{FFVt-1} * ffv_{t-1} + \beta_{AGE} * age_t + \beta_{MALE} * male + \beta_{NONWHITE} * nonWhite + \beta_{LOWINC} * lowinc + \beta_{MARRIED} * married + \beta_{EnvSMD} * EnvSMD\ ))$
$ALC_t = B(1,\ expit\ (Int_{ALC} + \beta_{ALCt-1} * alc_{t-1} + \beta_{AGE} * age_t + \beta_{MALE} * male + \beta_{NONWHITE} * nonWhite + \beta_{LOWINC} * lowinc + \beta_{MARRIED} * married\ ))$
$SMK_t = B(1,\ expit\ (Int_{SMK} + \beta_{SMKt-1} * smk_{t-1} + \beta_{AGE} * age_t + \beta_{MALE} * male + \beta_{NONWHITE} * nonWhite + \beta_{LOWINC} * lowinc + \beta_{MARRIED} * married\ ))$
$BMI_t = N(Int_{BMI} + \beta_{BMIt-1} * bmi_{t-1} + \beta_{SSBt-1} * ssb_{t-1} + \beta_{FFDt-1} * ffd_{t-1} + \beta_{MVPAt-1} * mvpa_{t-1} + \beta_{FFVt-1} * ffv_{t-1} + \beta_{AGE} * age_t + \beta_{MALE} * male + \beta_{NONWHITE} * nonWhite + \beta_{LOWINC} * lowinc + \beta_{MARRIED} * married\ , SD\_BMIt^2)$
$T2DM_t = B(1,\ expit\ (Int_{D2M} + \beta_{BMIt-1} * bmi_{t-1} + \beta_{BMI\_Ado} * bmi_{Ado} + \beta_{SSBt-1} * ssb_{t-1} + \beta_{FFDt-1} * ffd_{t-1} + \beta_{MVPAt-1} * mvpa_{t-1} + \beta_{FFVt-1} * ffv_{t-1} + \beta_{AGE} * age_t + \beta_{MALE} * male + \beta_{NONWHITE} * nonWhite + \beta_{LOWINC} * lowinc + \beta_{MARRIED} * married + \beta_{FamD2M} * famd2m\ ))$ *For those with* $T2DM_{t-1}$ *= 0*

Note that β represents a general notation for regression coefficients and is expected to differ across equations and age-groups (i.e., at birth, early childhood, middle childhood, adolescence, young adulthood and middle adulthood). Expit is the inverse function of the log-odds or logit function. EBF: Exclusive breastfeeding; FFD: Fast-food consumption; MVPA: Moderate-to-vigorous physical activity; SSB: Sugar-sweetened beverage consumption; FFV: Fresh fruit and vegetable consumption; SMK: Smoking; ALC: Alcohol consumption; EnvWLK: Environmental or neighborhood walkability; EnvPRK: Environmental or neighborhood park access; EnvSMD: Environmental or neighborhood supermarket density; EnvFFD: Environmental or neighborhood fast-food density; BMI: body mass index; T2DM: type 2 diabetes mellitus; Ado: Adolescence. T is an index of time

Table 12 Evaluation of the model calibration using the estimated R^2

Variable	R^2
Exclusive breastfeeding	NA
Physical activity	0.61
Fast food consumption	0.98
Fresh fruits and vegetables	0.78
Sugar-sweetened beverages	0.97
Body mass index	0.96
Smoking	NA
Alcohol	NA
Type 2 diabetes	NA

The R^2 for exclusive breastfeeding, smoking, alcohol, and type 2 diabetes could not be computed because of the low number of data points available.

References

1. Mortensen LH, Siegler IC, Barefoot JC, et al. (2006) Prospective associations between sedentary lifestyle and BMI in midlife. *Obes. (Silver Spring)* **14**, 1462–1471.
2. U.S. Census Bureau (2014) American Community Survey, 2010-2014 American Community Survey 5-year estimates. http://factfinder2.census.gov (accessed January 2016).
3. Center for Disease Control and Prevention (2012) *Breastfeeding Report Card — United States , 2012*. .
4. California Health Interview Survey (AskCHIS) (2016) 2009 California Health Interview Survey. http://ask.chis.ucla.edu/ (accessed January 2016).
5. World Health Organization (WHO) (2015) WHO BMI-for-age growth charts. *WHO*. World Health Organization; http://www.who.int/growthref/who2007_bmi_for_age/en/ (accessed September 2016).
6. Los Angeles County Department of Public Health Office of Health Assessment and Epidemiology (2016) Los Angeles Health And Nutrition Examination Survey (LAHANES 2011-2012). http://publichealth.lacounty.gov/ (accessed January 2016).
7. De Kroon ML, Renders CM, Buskermolen MP, et al. (2011) The Terneuzen Birth Cohort. Longer exclusive breastfeeding duration is associated with leaner body mass and a healthier diet in young adulthood. *BMC Pediatr* **11**, 33.
8. Li X-H, Lin S, Guo H, et al. (2014) Effectiveness of a school-based physical activity intervention on obesity in school children: a nonrandomized controlled trial. *BMC Public Health* **14**, 1282.
9. Aune D, Norat T, Leitzmann M, et al. (2015) Physical activity and the risk of type 2 diabetes: A systematic review and dose-response meta-analysis. *Eur. J. Epidemiol.* **30**, 529–542. Springer Netherlands.
10. Forshee RA, Anderson PA & Storey ML (2008) Sugar-sweetened beverages and body mass index in children and adolescents: A meta-analysis. *Am. J. Clin. Nutr.* **87**, 1662–1671.
11. Imamura F, O'Connor L, Ye Z, et al. (2015) Consumption of sugar sweetened beverages, artificially sweetened beverages, and fruit juice and incidence of type 2 diabetes: systematic review, meta-analysis, and estimation of population attributable fraction. *BMJ* **351**, h3576.
12. Bertoia ML, Mukamal KJ, Cahill LE, et al. (2015) Changes in Intake of Fruits and Vegetables and Weight Change in United States Men and Women Followed for Up to 24 Years: Analysis from Three Prospective Cohort Studies. *PLoS Med.* **12**, 1–20.
13. Wu Y, Zhang D, Jiang X, et al. (2015) Fruit and vegetable consumption and risk of type 2 diabetes mellitus: A dose-response meta-analysis of prospective cohort studies. *Nutr. Metab. Cardiovasc. Dis.* **25**, 140–147.
14. Pereira MA, Kartashov AI, Ebbeling CB, et al. (2005) Fast-food habits, weight gain, and insulin resistance (the CARDIA study): 15-year prospective analysis. *Lancet* **365**, 36–42.
15. Pan A, Sun Q & Bernstein A (2011) Red meat consumption and risk of type 2 diabetes: 3 cohorts of US adults and an updated meta-analysis. *Am.*, 1–9.
16. Llewellyn A, Simmonds M, Owen CG, et al. (2016) Childhood obesity as a predictor of morbidity in adulthood: A systematic review and meta-analysis. *Obes. Rev.* **17**, 56–67.
17. Moore L V., Diez Roux A V., Nettleton JA, et al. (2008) Associations of the local food

environment with diet quality - A comparison of assessments based on surveys and geographic information systems. *Am. J. Epidemiol.* **167**, 917–924.

18. Moore L V., Diez Roux A V., Nettleton JA, et al. (2009) Fast-Food consumption, diet quality, and neighborhood exposure to fast food. *Am. J. Epidemiol.* **170**, 29–36.
19. Brown SC, Pantin H, Lombard J, et al. (2013) Walk score: Associations with purposive walking in recent cuban immigrants. *Am. J. Prev. Med.* **45**, 202–206. Elsevier.
20. Giles-Corti B, Broomhall MH, Knuiman M, et al. (2005) Increasing walking: How important is distance to, attractiveness, and size of public open space? *Am. J. Prev. Med.* **28**, 169–176.
21. Wolch J, Wilson J & Fehrenbach J (2005) Parks and Park Funding in Los Angeles: An Equity-Mapping Analysis. *Urban Geogr.* **26**, 4–35.
22. Walls and Associates/Your Economy (2016) National Establishement Time-Series (NETS) Database by Walls & Associates. http://143.235.14.134/our-databases.iegc#NETS (accessed January 2016).
23. National Center for Health Statistics CDC (2015) National Health and Nutrition Examination Survey (NHANES) - Questionnaires, Datasets, and Related Documentation. http://www.cdc.gov/nchs/nhanes/nhanes_questionnaires.htm (accessed January 2015).
